# Delayed spontaneous closure of traumatic macular hole in a 66-year-old patient – role of optical coherence tomography follow-up

**DOI:** 10.3205/oc000168

**Published:** 2020-08-28

**Authors:** Piergiacomo Grassi, Alberto Salicone

**Affiliations:** 1 Department of Ophthalmology, Royal Hallamshire Hospital, Sheffield University Hospitals NHS Foundation Trust, Sheffield, United Kingdom; 2 Unit of Ophthalmology, San Leonardo Hospital, Castellammare di Stabia NA, Italy

**Keywords:** epiretinal membrane, macular hole, optical coherence tomography, pars-plana vitrectomy, spontaneous closure, traumatic macular hole

## Abstract

**Objective:** To report a case of delayed spontaneous closure of traumatic macular hole at 5 months in a 66-year-old man. Traumatic macular holes generally do not close spontaneously after 4 months and over 30 years of age.

**Methods:** A 66-year-old man presented with central blurred vision and metamorphopsia in his right eye for 3 weeks after previous ocular blunt trauma occurring 2 months earlier. Best corrected visual acuity was 6/36 in his right eye, fundus examination and OCT revealed right traumatic macular hole.

**Results:** 4 weeks later, best corrected visual acuity was 6/18, OCT showed initial reattachment of traumatic macular hole margins. 8 weeks later, best corrected visual acuity improved to 6/9, OCT showed almost complete reattachment of the margins, residual outer retinal defect being still present. At 12 weeks after initial presentation, best corrected visual acuity was 6/6, OCT showed normal neuroretinal profile.

**Conclusion:** Clinical monitoring of traumatic macular holes might be performed up to 5 months even in patients >30 years before considering surgery.

## Introduction

Macular holes (MH) are retinal defects of 200–550 µm in diameter that involve the fovea and are well-known complications of ocular contusion injury occurring in 1.4% of closed globe injuries and 0.15% of open globe injuries [1]. Sports-related accidents are the major cause of ocular blunt trauma, thus the higher frequency of traumatic macular holes (TMH) in younger patients [2]. Typical signs and symptoms include acute visual loss, blurred and distorted vision and central scotoma, and immediate visual loss after injury is probably due to retinal dehiscence on concussion, whereas delayed visual loss is likely to indicate a secondary event of vitreoretinal interface changes. Optical coherence tomography (OCT) allows an objective diagnosis and monitoring of TMH and confirms its resolution, avoiding a never-without-risks surgery [3]. Small-gauge pars-plana vitrectomy (PPV) and fluid-gas exchange is the current surgical management for TMH repair. However, spontaneous closure (SC) of MH is not rare, especially among young patients, and occurs in approximately 50% of cases, but rarely after 4 months and over 30 years of age [4], [5].

An unusual case of TMH in a 66-year-old patient which spontaneously closed after 5 months with no need for further treatment is described.

## Case description

**Figure 1:**
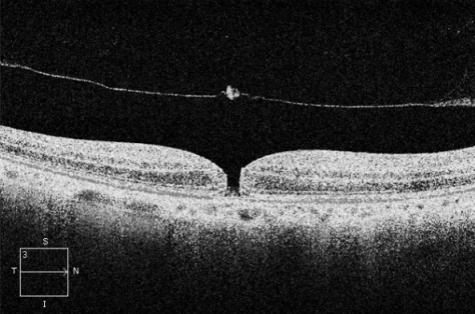
OCT horizontal scan at presentation, showing TMH with a diameter of 232 µm, irregular retinal profile, absence of epiretinal membrane, delamination in the outer nuclear layer, Bruch’s membrane attached to the retinal pigment epithelium, initial separation of the posterior hyaloid membrane from inner retina with overlying retinal operculum

**Figure 2:**
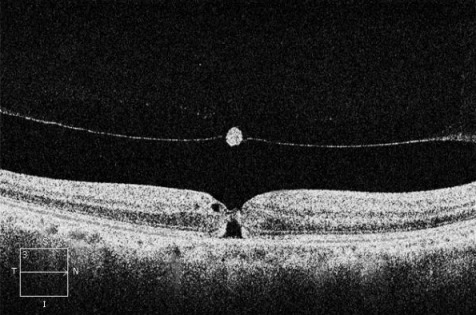
OCT horizontal scan at 4 weeks follow-up, showing initial reattachment of the TMH margins

**Figure 3:**
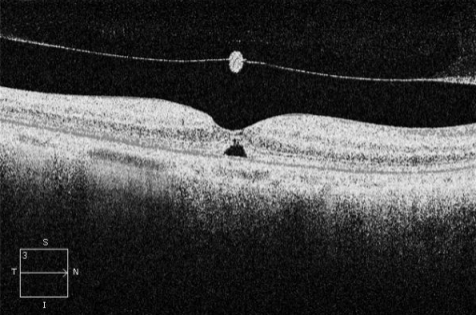
OCT horizontal scan at 8 weeks follow-up, showing almost complete reattachment of the margins of TMH, residual outer retinal defect being still present

**Figure 4:**
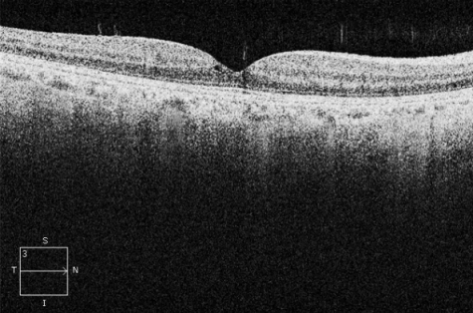
OCT horizontal scan at 12 weeks follow-up, showing normal neuroretinal profile and thickness and complete closure of the TMH

A 66-year-old man attended Eye Casualties with central blurred vision and metamorphopsia in his right eye for 3 weeks after previous ocular blunt trauma occurring 2 months earlier. The patient was in good health taking no medications, and had no personal or family history of eye diseases, hypertension, diabetes mellitus, or dyslipidemias. Best corrected visual acuity (BCVA) was 6/36 in his right eye with positive Amsler grid testing (AGT). Fundus examination of his right eye showed loss of foveolar light reflex and a round small lesion with red base at the centre of his right macula. OCT confirmed the presence of TMH with a diameter of 232 µm, absence of epiretinal membrane (ERM), delamination in the outer nuclear layer and initial separation of the posterior hyaloid membrane from the inner retina with overlying retinal operculum (Figure 1 (Fig. 1)). Small full thickness MH was diagnosed according to the International Vitreomacular Traction Study Classification System, no topical/systemic medical treatments were started. 4 weeks later, BCVA in the patient’s right eye was 6/18, OCT showed initial reattachment of the TMH margins (Figure 2 (Fig. 2)). 8 weeks later, right BCVA improved to 6/9 with slightly positive AGT. OCT showed almost complete reattachment of the margins of TMH, residual outer retinal defect still being present (Figure 3 (Fig. 3)). At 12 weeks after initial presentation, BCVA in the patient’s right eye was 6/6 with negative AGT. OCT showed normal neuroretinal profile and thickness (Figure 4 (Fig. 4)), BCVA and OCT remained unchanged at 8 months examination.

## Discussion

The precise mechanism of TMH formation is unclear, and the time of appearance is highly variable (directly after the ocular trauma up to 1 month), suggesting multiple mechanisms of formation. Yamashita et al. proposed two pathogenic mechanisms for TMH formation. The first is the most common, posterior vitreous detachment (PVD) is not involved, and the origin of post-contusive TMH is associated with sudden deformation of the eyeball and anterior-posterior compression with retinal stretching. These forces cause transverse tractional retinal breakdown of the macula, MH is then formed almost immediately after the trauma and causes immediate visual loss due to primary dehiscence of the fovea. In contrast, in the second mechanism visual loss is more gradual, perifoveal PVD plays a role and suggests that vitreoretinal foveal traction is responsible for the slow formation of a TMH (several days or even weeks after the injury). The gripping power forces are no longer transverse, but anterior-posterior [5]. However, in older patients, posterior vitreous is usually detached, making TMH generally less frequent in older patients [5].

There are no clinical studies that demonstrate the natural history of TMH. The need to undergo surgical repair and the timing of possible intervention are not well defined [1], and SC of TMH has been previously reported [1], [4], [6], [7]. Miller et al. found a 39.3% SC rate of TMH in a median of 5.7 weeks; the 39.3% of their cases underwent PPV (5 eyes out of 11 (45%) were successfully closed), and BCVA improved significantly in both eyes with TMH closed either spontaneously (P>.01) or via PPV (P=.04) [7]. A meta-analysis of surgical outcomes in all published reports of PPV for TMH found a successful closure rate of 83% [7]. Clinical observation through OCT allows to monitor the TMH objectively and to confirm its resolution, avoiding the risks related to surgery [3]. However, permanent macular structural changes, TMH enlargement and retinal detachment may occur during observation. Patients <30 years old without involvement of the posterior hyaloid, no pre-existing PVD, small size of TMH and its reduction in size during the first weeks after trauma seem to be associated with SC, whereas TMH enlargement over the first weeks may indicate a poor prognosis for SC and merit further consideration of surgery [1], [7]. Our case of TMH experienced delayed symptoms and, in spite of the patient’s age, complete SC after 5 months from initial ocular trauma with no need of further treatment, suggesting a perifoveal PVD mechanism and providing a compelling argument for a systematic clinical monitoring of TMH within at least 5 months after trauma even in patients >30 years, contrary to what most of the literature supports [7]. Since OCT allows an objective monitoring and provides objective data about MH evolution and confirms their resolution, clinical monitoring should be based on OCT and should be performed for 3 to 5 months before considering surgery [3]. This may not be applicable for a subset of TMH with severe traction on the edges, where an earlier surgical intervention may be suggested by the increase in size and enlargement of TMH from presentation to last follow-up examination since it indicates a poor prognosis for SC [1]. Importantly, an intact ellipsoid zone in closed holes seems to correlate with better final BCVA regardless of how TMH closure occurs (observation or vitrectomy). Surgery within 6 months of the onset of TMH may not affect the final outcome, and SC mainly occurs during the few months after trauma. 

## Conclusion

A close clinical and instrumental monitoring post TMH without ERM and poor vitreal adherence might be appropriate in order to verify a possible SC [8], even in patients over 30 years of age and after several weeks from ocular trauma.

## Notes

### Authors’ contributions

- Concept and design of the study: Alberto Salicone
- Acquisition of data: Alberto Salicone, Piergiacomo Grassi
- Analysis and interpretation of data: Piergiacomo Grassi
- Writing of original draft: Alberto Salicone, Piergiacomo Grassi
- Critical revision, review and editing: Piergiacomo Grassi

### Competing interests

The authors declare that they have no competing interests.
